# Red Seaweeds *Sarcodiotheca gaudichaudii* and *Chondrus crispus* down Regulate Virulence Factors of *Salmonella* Enteritidis and Induce Immune Responses in *Caenorhabditis elegans*

**DOI:** 10.3389/fmicb.2016.00421

**Published:** 2016-03-31

**Authors:** Garima Kulshreshtha, Tudor Borza, Bruce Rathgeber, Glenn S. Stratton, Nikhil A. Thomas, Alan Critchley, Jeff Hafting, Balakrishnan Prithiviraj

**Affiliations:** ^1^Department of Environmental Sciences, Faculty of Agriculture, Dalhousie UniversityTruro, NS, Canada; ^2^Acadian Seaplants LimitedDartmouth, NS, Canada; ^3^Department of Plant and Animal Sciences, Faculty of Agriculture, Dalhousie UniversityTruro, NS, Canada; ^4^Department of Microbiology and Immunology, Faculty of Medicine, Dalhousie UniversityHalifax, NS, Canada

**Keywords:** *Salmonella* enteritidis, virulence factors, *Chondrus crispus*, *Sarcodiotheca gaudichaudii*, *Caenorhabditis elegans*, immune response

## Abstract

Red seaweeds are a rich source of unique bioactive compounds and secondary metabolites that are known to improve human and animal health. *S*. Enteritidis is a broad range host pathogen, which contaminates chicken and poultry products that end into the human food chain. Worldwide, *Salmonella* outbreaks have become an important economic and public health concern. Moreover, the development of resistance in *Salmonella* serovars toward multiple drugs highlights the need for alternative control strategies. This study evaluated the antimicrobial property of red seaweeds extracts against *Salmonella* Enteritidis using the *Caenorhabditis elegans* infection model. Six red seaweed species were tested for their antimicrobial activity against *S*. Enteritidis and two, *Sarcodiotheca gaudichaudii* (SG) and *Chondrus crispus* (CC), were found to exhibit such properties. Spread plate assay revealed that SG and CC (1%, w/v) significantly reduced the growth of *S*. Enteritidis. Seaweed water extracts (SWE) of SG and CC, at concentrations from 0.4 to 2 mg/ml, significantly reduced the growth of *S*. Enteritidis (log CFU 4.5–5.3 and log 5.7–6.0, respectively). However, methanolic extracts of CC and SG did not affect the growth of *S*. Enteritidis. Addition of SWE (0.2 mg/ml, CC and SG) significantly decreased biofilm formation and reduced the motility of *S*. Enteritidis. Quantitative real-time PCR analyses showed that SWE (CC and SG) suppressed the expression of quorum sensing gene *sdiA* and of Salmonella Pathogenesis Island-1 (SPI-1) associated genes *sipA* and *invF*, indicating that SWE might reduce the invasion of *S*. Enteritidis in the host by attenuating virulence factors. Furthermore, CC and SG water extracts significantly improved the survival of infected *C. elegans* by impairing the ability of *S*. Enteritidis to colonize the digestive tract of the nematode and by enhancing the expression of *C. elegans* immune responsive genes. As the innate immune response pathways of *C. elegans* and mammals show a high degree of conservation, these results suggest that these SWE may also impart beneficial effects on animal and human health.

## Introduction

Food-borne pathogen *Salmonella enterica* subsp. *enterica* serovar Enteritidis (*S*. Enteritidis) is the world's leading cause of egg associated salmonellosis in humans (Sheela et al., 2003; Govaris et al., 2010). *S*. Enteritidis is a broad range host pathogen carried by chicken and poultry products to human food chain. In humans, *Salmonella* infection causes food poisoning and intestinal infections associated with mucosal inflammation and diarrhea leading, in some cases, to mortality (Yim et al., 2010). Worldwide, *Salmonella* outbreaks have become an important public health and economic concern (World Health Organization, 2014). World Health Organization global *Salmonella* survey program estimated that a number of 500–2000 deaths occur each year (Betancor et al., 2010; Yim et al., 2010). In 2003, 12.7% of all *Salmonella* cases were due to *S*. Enteritidis; in 2009 the percentage increased to 32.1% (Nesbitt et al., 2012). In Canada and United States, each year approximately 1.4 million people are infected with non-typhoid *Salmonella* serotype (Nesbitt et al., 2012; Middleton et al., 2014). The national enteric disease surveillance report from 2011 indicated that in United States *S*. Enteritidis is the most dominant serotype from clinical and non-clinical sources (Center for Disease Control and Prevention, 2012), while in Canada is one of the top three non-typhoidal serovars (Galanis et al., 2012).

In chickens, *S*. Enteritidis colonizes the gastrointestinal tract, from where infection can extend to organs such as the ovaries and the oviduct to eventually localize inside the egg and embryo (Guard-Petter, 2001). The ability of *S*. Enteritidis to establish persistent infection in avian tissues including egg is responsible for its invasion into the human food chain (Revolledo et al., 2009; Yim et al., 2010).

Upon consumption of contaminated water or food, *S*. Enteritidis recognizes and adhere to the host epithelium (Pontier-Bres et al., 2012). Bacteria penetrate the intestinal epithelium by suppressing signal transduction pathways leading to cytoskeleton rearrangement into the host cell. This is followed by the delivery of the effector proteins, which suppress the immune response of the host, establishing a persistent infection (Groisman and Mouslim, 2000; Brown et al., 2005). The survival capabilities of *S*. Enteritidis are enhanced by quorum sensing and by the formation of biofilms on a variety of biotic and abiotic surfaces (De Kievit and Iglewski, 2000; Parker and Guard-Petter, 2001; Prouty et al., 2002; Brossard and Campagnari, 2012).

*Salmonella* infection can be fatal in immunocompromised patients if not treated with antibiotics. Fluoroquinolones and cephalosporin are most commonly used antibiotics to treat infections caused by *Salmonella* serovars (Baucheron et al., 2004). However, the development of resistance in *Salmonella* serovars toward multiple drugs highlights the urgent need for alternative strategies to control this pathogen (Acheson and Hohmann, 2001). Previously, bacteriophages, antimicrobial peptides, and essential oils have been used or investigated as alternatives to antibiotics (Fratamico and Cooke, 1996; Joerger, 2003).

*Salmonella* also infects the nematode *Caenorhabditis elegans*, a widely used model organism (Aballay et al., 2000; Aballay and Ausubel, 2002; Sifri et al., 2005). Several studies have shown that bacterial pathogens such as *Pseudomonas aeruginosa, Staphylococcus aureus, Vibrio* sp., *Salmonella* Typhimurium, *E. coli* 0157:H7, and *Enterococcus faecalis* have similar pathogenic mechanisms in nematodes and higher animals (Aballay et al., 2000; Aballay and Ausubel, 2002; Breger et al., 2007). For example, the pathosystem *C. elegans—E. faecalis* has been used as high throughput model to screen compounds with potential anti-infective and anti-microbial properties, applicable to cure infections in higher animals and humans (Moy et al., 2009). Additionally, Tenor et al. (2004) have shown that *C. elegans* is an attractive model to study the interaction between *Salmonella* effector protein and host innate immunity because there is a significant overlap between virulence factors of *Salmonella* required for both, nematode and human pathogenesis. *C. elegans* react to *Salmonella* infection by activating the innate immune response through the p38 mitogen-activated protein kinase (PMK-1) and subsequently by synthesizing antimicrobial peptides, mechanisms that are similar to immune responses in humans (Aballay et al., 2003; Alegado and Tan, 2008).

Red seaweeds are a rich source of lipids, polysaccharides, proteins, bioactive compounds and of secondary metabolites such as polyphenols as well as of minerals, which impart several health benefits (Pujol et al., 2002; Bansemir et al., 2004; Yuan et al., 2005; Lins et al., 2009; Gómez-Ordóñez et al., 2012; Souza et al., 2012). Edible red seaweeds, *Sarcodiotheca gaudichaudii* and *Chondrus crispus*, are abundant along the coasts of the eastern Pacific Ocean and of western Atlantic Ocean (Gabrielson, 1982; Guiry and Guiry, 2016) and certain strains of red seaweeds are commercially cultivated in land (Hafting et al., 2012). Red seaweeds have been recently explored as potential sources of products with antimicrobial properties. The main polysaccharides in these seaweeds, the carrageenans, were shown to have antiviral properties as well as antitumor, anticoagulant and immunomodulatory effects (Campo et al., 2009; de Jesus Raposo et al., 2015). It has been shown that other red seaweed compounds such as derived brominated furanones reduced swimming motility, flagellar biosynthesis in *Salmonella* serovar Typhimurium and showed biofilm inhibiting activities (Janssens et al., 2008). Recently, components of cultivated red seaweeds have been shown to improve the immune response of *C. elegans* to *Pseudomonas aeruginosa* (PA-14) through the induction of PMK-1 and Daf-2/daf-16 insulin signaling pathways (Liu et al., 2013). Furthermore, enzymatic extracts of *C. crispus* were identified as effective against HSV-1 virus, indicating potential antiviral activity of sulphated polysaccharides in the extracts (Kulshreshtha et al., 2015). In another study, feed supplementation with *S. gaudichaudii* and *C. crispus* reduced the prevalence of pathogenic bacteria such as *Clostridium perfringens* in the chicken gut while the relative abundance of beneficial bacteria such as *Bifidobacterium longum* and *Streptococcus salivarius* was found to be increased (Kulshreshtha et al., 2014). Here, we report the effects of water extracts from *S. gaudichaudii* and *C. crispus* on *Salmonella* Enteritidis using the *C. elegans* infection model. In addition, we also examined the effects of water extracts on biofilm formation, motility, quorum sensing signaling and virulence factors in *S*. Enteritidis. Red seaweeds are a rich source of lipids, polysaccharides, proteins, bioactive compounds and of secondary metabolites such as polyphenols as well as of minerals, which impart several health benefits (Pujol et al., 2002; Bansemir et al., 2004; Yuan et al., 2005; Lins et al., 2009; Gómez-Ordóñez et al., 2012; Souza et al., 2012). Edible red seaweeds, *Sarcodiotheca gaudichaudii* and *Chondrus crispus*, are abundant along the coasts of the eastern Pacific Ocean and of western Atlantic Ocean (Gabrielson, 1982; Guiry and Guiry, 2016) and certain strains of red seaweeds are commercially cultivated in land (Hafting et al., 2012). Red seaweeds have been recently explored as potential sources of products with antimicrobial properties. The main polysaccharides in these seaweeds, the carrageenans, were shown to have antiviral properties as well as antitumor, anticoagulant and immunomodulatory effects (Campo et al., 2009; de Jesus Raposo et al., 2015). It has been shown that other red seaweed compounds such as derived brominated furanones reduced swimming motility, flagellar biosynthesis in *Salmonella* serovar Typhimurium and showed biofilm inhibiting activities (Janssens et al., 2008). Recently, components of cultivated red seaweeds have been shown to improve the immune response of *C. elegans* to *Pseudomonas aeruginosa* (PA-14) through the induction of PMK-1 and Daf-2/daf-16 insulin signaling pathways (Liu et al., 2013). Furthermore, enzymatic extracts of *C. crispus* were identified as effective against HSV-1 virus, indicating potential antiviral activity of sulphated polysaccharides in the extracts (Kulshreshtha et al., 2015). In another study, feed supplementation with *S. gaudichaudii* and *C. crispus* reduced the prevalence of pathogenic bacteria such as *Clostridium perfringens* in the chicken gut while the relative abundance of beneficial bacteria such as *Bifidobacterium longum* and *Streptococcus salivarius* was found to be increased (Kulshreshtha et al., 2014). Here, we report the effects of water extracts from *S. gaudichaudii* and *C. crispus* on *Salmonella* Enteritidis using the *C. elegans* infection model. In addition, we also examined the effects of water extracts on biofilm formation, motility, quorum sensing signaling and virulence factors in *S*. Enteritidis.

## Materials and methods

### Preparation of seaweed extract (SWE)

**Figure 1:**
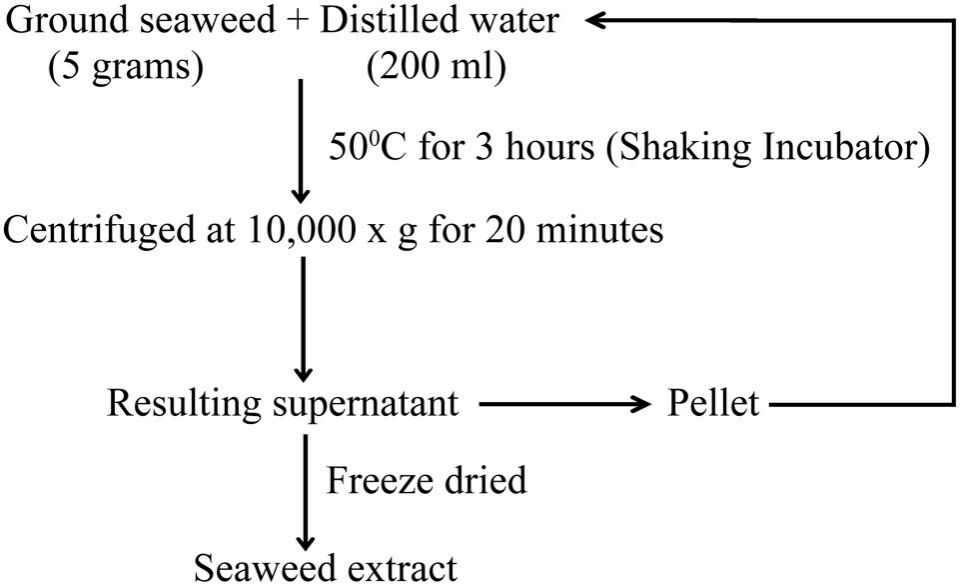
**The procedure for the extraction of red seaweed water extract**.

Red seaweeds (*Chondrus crispus, Gymnogongrus devoniensis, Palmaria palmata, Sarcodiotheca gaudichaudii, Solieria chordalis*, and *Sarcodiotheca* spp.) were provided by Acadian Seaplants Limited, Nova Scotia, Canada. The extraction procedure is summarized in Figure 1. Sun dried seaweeds were ground to a fine powder using a coffee grinder. Seaweed water extracts (SWE) were prepared by adding 5 g of algal powder to 20 ml distilled water (DW); the slurry was incubated at 50°C for 3 h under shaking condition at 140 rpm (New Brunswick Scientific, Enfield, CT, US). After centrifugation at 10,000 g for 15 min. the supernatant was recovered and the residual pellet was re-extracted three times. The resulting supernatants were pooled and freeze dried (Freeze dryer, Thermo Fisher Scientific Inc., US). The stock solution of 10 mg/ml SWE was prepared by dissolving the soluble freeze dried extract in distilled water and used to prepare the 0.2, 0.4, 0.8, 1, and 2 mg/ml dilutions of SWE used in the experiments.

### Bacterial strains, growth condition, and *C. elegans*

Nalidixic acid resistant stain of *S*. Enteritidis was provided by Laboratory for Foodborne Zoonoses, Public Health Agency of Canada, Guelph, Ontario. Half strength tryptic soy agar (TSA) medium (Difco) supplemented with nalidixic acid at a concentration of 32 μg/ml was used for bacterial growth (Ebers et al., 2009). *C. elegans* strain Bristol N2 was maintained on modified nematode growth medium (0.35% peptone instead of 0.25%) at 20°C seeded with *Escherichia coli* OP50 as food source (Aballay and Ausubel, 2001). Bacterial strains were grown overnight at 37°C and were equilibrated to OD_600_ = 0.1 (1 × 10^8^ cells/ml) to maintain uniform bacterial cell count. All experiments were repeated three times with six biological replicates.

### Bacterial inhibition test

Seaweeds were screened for antimicrobial activity against *S*. Enteritidi*s* by spread plate technique (Buck and Cleverdon, 1960). Hundred microliters of fresh overnight culture of *S*. Enteritidis (OD_600_ = 0.1) was spread plated on the TSA plates containing ground seaweed (1% w/v). The plates were incubated at 37°C for 24 h and log CFU was calculated after bacteria were enumerated. Seaweeds showing maximum inhibition were selected for preparation of seaweed extract.

#### Broth inoculation method

*S*. Enteritidis suspension (100 μl with an OD_600_ = 0.1) was added to 10 ml of tryptic soy broth containing 200, 400, or 800 μg/ml of SWE. Culture tubes were incubated at 37°C for 24 h. The growth of *S*. Enteritidis was measured spectrophotometrically at OD_600_ and bacteria were enumerated after serially diluting the treatments and bacterial dilution plating on TSA plates.

#### Agar well diffusion method

Antimicrobial activity of SWE was also evaluated by agar well diffusion assay with some modification (Bennett et al., 1966). Twenty milliliters of TSA (45°C) was poured into 15 cm sterile Petri dishes and *S*. Enteritidis was spread plated. Ten millimeter wells were bored using a sterile cork borer and known concentrations of SWE were added into the wells in the plates. The plates were incubated for 24 h at 37°C and antimicrobial activity was measured using vernier caliper to determine the zone of growth inhibition. H_2_O was used as negative control.

### Effect of SWE on bacterial motility—swimming and swarming

Ability of SWE to alter bacterial motility was tested as described by Rashid and Kornberg (2000) with some modifications (Difco bacteriological agar instead of agarose). Single colony of bacteria from overnight grown culture was spotted using a sterile toothpick on swim plates or on swarm plates containing known concentration of SWE (200 μg/ml). All plates were sealed with parafilm to prevent dehydration and swim plates were incubated at 30°C for 14–15 h while swarm plates for 24 h.

### Biofilm formation assay

Overnight grown *S*. Enteritidis culture was diluted 1:100 in tryptic soy broth containing known concentration of SWE (200 μg/ml). Two hundred μl of the aliquot was dispensed into 96 wells polyvinyl chloride microtitre plates. The plates were incubated statically at 28°C for 24 h. Biofilm formation was quantified by staining the wells with 20 μl of crystal violet (CV) (0.14% (w/v) in water) at room temperature for 20 min. The wells were washed three times in distilled water to remove excess CV. CV stained cells were eluted with 95% ethanol and optical density was measured at OD_600_.

### Effect of SWE on expression of virulence and quorum sensing related genes

For gene expression analysis, *S*. Enteritidis with an initial OD_600_ of 0.1 was cultured at 37°C TSB in the presence and absence (control) of SWE with shaking at 160 rpm. Bacterial cells were harvested by centrifugation at 12,000 g for 10 min. Total RNA was extracted using Trizol (Invitrogen) as described by the manufacturer. The RNA was quantified by NanoDrop ND-2000 spectrophotometer (NanoDrop Technologies Wilmington, DE) and the quality was assessed by agarose gel electrophoresis. RNA from each biological replicate was used for cDNA synthesis using the High Capacity cDNA reverse transcription kit (Applied Biosystems). The relative transcript levels of quorum sensing, virulence, and flagella associated genes were quantified using StepOnePlus Real time PCR (Applied Biosystems, ON, Canada). The 10 μl reaction mix contained 2 ng of cDNA, 5 μl Promega GoTaq SYBR green master mix (Promega North America, Madison, WI, USA) and 300 nM of gene specific primers (Supplementary Table 1). *16SrRNA* and *tufA* genes were used as internal control and the relative expression levels were calculated using the ΔΔCt method.

### *C. elegans* killing assay

Modified nematode growth medium was used to establish *C. elegans-S*. Enteritidis pathosystem as described by Aballay et al. (2000). *C. elegans* killing assay was conducted by two methods as described below:

*Incorporating SWE into the media:* Treatment plates were prepared by supplementing SWE to nematode growth media (NGM) to a final concentration of 200, 400, and 800 μg/ml. *C elegans* population was synchronized by placing adult nematodes on NGM plates to lay egg for 4–6 h. Eggs were incubated for 2 days at 20 ± 2°C to ensure uniform adult population. Thirty to forty synchronized L4 nematodes were used for each assay. Heat killed *S*. Enteritidis and *E. coli* OP50 were used as control and 70 μM fluorodeoxyuridine (FuDR) was used to prevent the development of progeny. The plates were incubated at 25°C and scored for live vs. dead worms every 24 h. A worm was considered dead when it failed to respond to plate tapping or a gentle touch with a platinum wire. Worms killed as a result of being stuck to the wall of the plate were excluded from the analysis. Nematodes were subjected to a combination of three pre-treatments with SWE to target virulence of bacteria and immune response of *C elegans* as described below:

- Pre-treatment of bacteria with SWE: Synchronized worms were infected with *S*. Enteritidis grown overnight on NGM plates containing 200, 400, 800 μg/ml of SWE to test its efficacy in reducing bacterial virulence.
- Pre-treatment of nematodes with SWE: Synchronized populations of worms were maintained on NMG plates from egg stage containing 200, 400, 800 μg/ml of SWE. Pre-treated L4 nematodes were transferred to *S*. Enteritidis treatment plates.
- Pre-treatment of bacteria and nematodes with SWE: Synchronized worms from egg stage maintained on NGM plated were infected with *S*. Enteritidis grown overnight on NGM plates containing 200, 400, 800 μg/ml of SWE.

*Adding SWE over the media: S*. Enteritidis was grown on modified NGM plates and 200, 400, 800 μg/ml of SWE was added over the media along with food source. The killing assay was performed with three combination of pre-treatment as described above.

### *S*. Enteritidis colonization assay of *C. elegans* gut

*S*. Enteritidis count of from C. elegans gut was determined according to the modified method previously described by Prithiviraj et al. (2005). For each replicate, six adult C. elegans were picked from the treatment plates and transferred into a 1.5 ml microfuge tube containing 500 μL of M9 buffer supplemented with 20 μg/ml gentamicin and washed three times to remove bacteria from C. elegans surface. The nematodes were disrupted in a microfuge tube containing 50 μL of M9 medium with 1% Triton X-100 using a microfuge pestle. The resulting slurry was serially diluted and plated on TSA medium and the number of CFU was counted.

### Effect of SWE on expression of immune response genes in *C. elegans*

Treatment plates were prepared by supplementing SWE to NGM to a final concentration of 400 μg/ml. *C. elegans* were infected with *S*. Enteritidis and approximately 100 worms per treatment were harvested 5 days after exposure to *S*. Enteritidis. There were the four conditions used in the experiment: (1) *C. elegans* fed on SWE, (2) *C. elegans* fed on *S*. Enteritidis, (3) *C. elegans* fed on SWE and on *S*. Enteritidis, and (4) *C. elegans* fed on heat killed *E. coli* OP50. Worms were transferred into 1.5 ml microfuge tubes and washed three times in M9 buffer to eliminate excess bacteria. Excess buffer was pipetted out and total RNA was extracted using Trizol (Invitrogen) following manufacturer's protocol. RNA quality and quantity determination, cDNA synthesis and quantitative real time PCR were performed as previously described. The immune responsive genes specific primers used for these experiments are listed in Supplementary Table 2. Relative expression levels were determined by ΔΔCt method and *ama-1* was used as a reference gene while heat killed *E. coli* OP50 samples were used as control.

### Statistical analysis

A completely randomized design was followed to analyze effects of application method, concentration, and antimicrobial assays. All experiments were performed three times with at least three biological replicates. Data was analyzed using ANOVA one-way analysis of variance with a *P*-value of 0.05 using the statistical software Minitab 17 (Minitab Inc., PA, USA) and SAS, version 9.4 for Windows (SAS Institute Inc., NC, USA). If significant main effects were found with ANOVA, the Tukey's procedure was used to compare differences among the least-square means. The standard deviation (SD) was reported with the mean. Differences were considered significant when *P* was < 0.05.

## Results

### Seaweed extracts reduces the growth of *Salmonella* enteritidis

**Figure 2:**
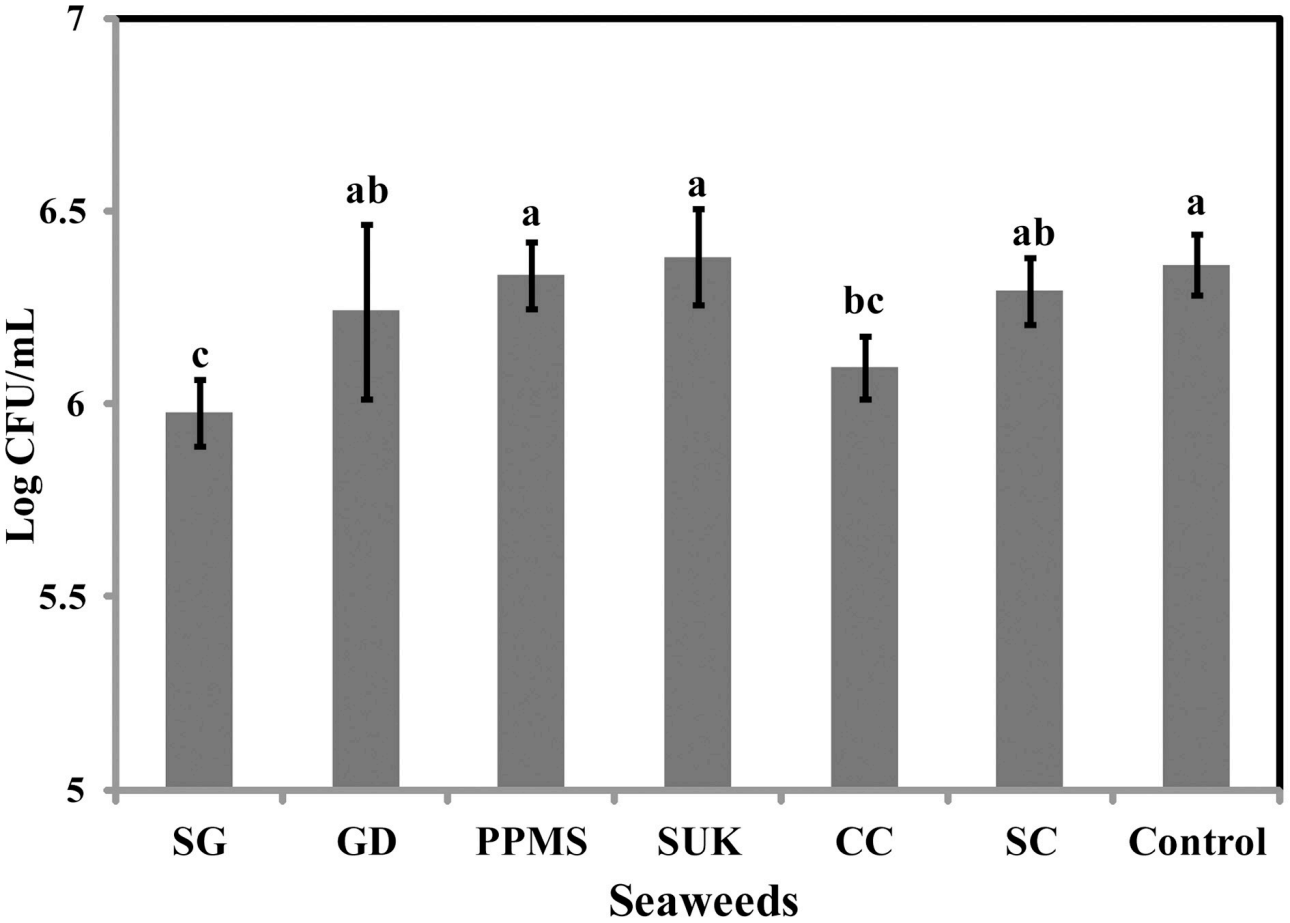
**Effect of red seaweeds on the growth of *Salmonella* Enteritidis growth**. Six red seaweed species *Chondrus crispus* (CC), *Gymnogongrus devoniensis* (GD), *Palmaria palmate* (PPMS), *Sarcodiotheca gaudichaudii* (SG), *Solieria chordalis* (SC), and *Sarcodiotheca spp* (SUK) were tested against *S*. Enteritidis. A hundred μl of fresh overnight culture was spread plated on the TSA plates containing ground seaweed (1% w/v). Log CFU/mL was calculated after incubating the plates at 37°C for 24 h. Values with different superscript letters (Tukey multiple mean comparison) are significantly different (one-way Anova; *p* < 0.05). Values represent mean ± standard deviation from three independent experiments (*n* = 9).

**Figure 3:**
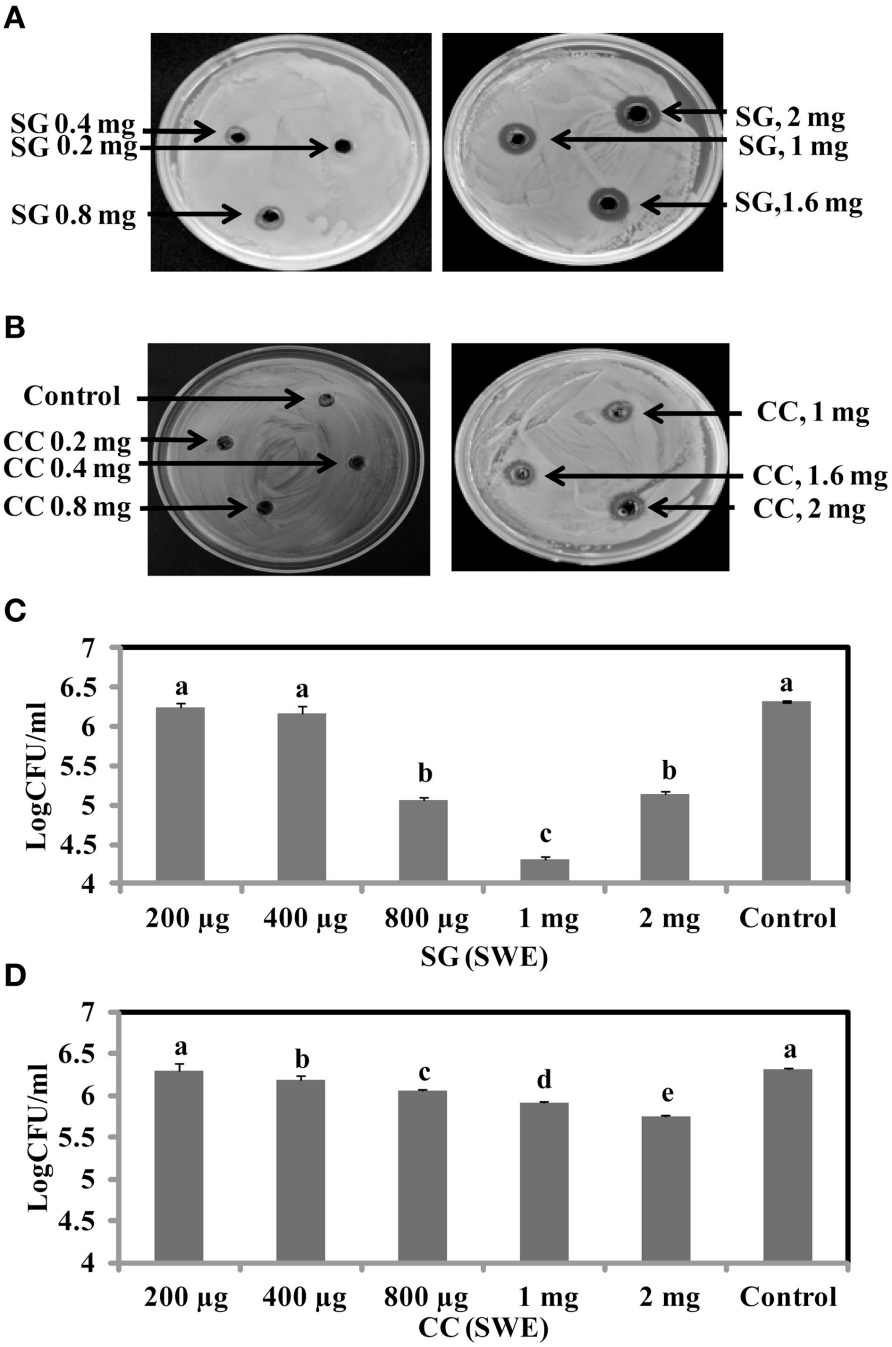
**Antimicrobial activities of red seaweeds**. *Chondrus crispus* (CC) and *Sarcodiotheca gaudichaudii* (SG) were tested at different concentration against *S*. Enteritidis by agar well diffusion method and liquid culture broth inoculation method. Solid agar showing the zone of growth inhibition at different concentration of **(A)** SG **(B)** CC. Liquid culture showing the bacterial colony count at different concentrations of **(C)** CC extract and **(D)** SG extract. Values with different superscript letters (Tukey multiple mean comparison) are significantly different (one-way Anova; *p* < 0.05). Values represent mean ± standard deviation from three independent experiments (*n* = 9).

To identify the antimicrobial potential of red seaweeds, six selected powdered seaweeds were amended to TSA and tested against *S*. Enteritidis by spread plate technique. At a concentration of 1% w/v only *Sarcodiotheca gaudichaudii* (SG) and *Chondrus crispus* (CC) significantly reduced growth of *S*. Enteritidis (Figure 2); therefore these two seaweeds were selected for all further assays. Water and methanol extracts were prepared from SG and CC and were tested against *S*. Enteritidis by well diffusion and broth inoculation methods. In well diffusion plate method SG (water extract) inhibited the growth of *S*. Enteritidis in a concentration dependent manner, i.e., lower at 400 μg/ml mg/ml and higher, at 2 mg/ml with the zone of inhibition increasing from 3 to 13 mm (Figure 3A and Supplementary Table 3). For CC, concentrations of 1 mg/ml and higher (1.6 and 2 mg/ml) were required to generate a clear zone of growth inhibition (4–9 mm) (Figure 3B and Supplementary Table 3). Organic extracts of both SG and CC did not display any antimicrobial activity therefore only water extract was used for all further experiments.

The antimicrobial activity of both SG and CC water extract was further verified by liquid culture inhibition test. SG at concentrations of 200 and 400 μg/ml and CC at a concentration of 200 μg/ml did not affect the growth of *S*. Enteritidis. However, in the case of SG, the bacterial titers of *S*. Enteritidis were significantly reduced by concentrations of 0.8, 1, and 2 mg/ml (log CFU 4.5–5.3, *p* < 0.05) (Figure 3C). Similarly, for CC, higher concentrations (0.4, 0.8, 1, and 2 mg/ml) significantly reduced the growth of *S*. Enteritidis (log 5.7–6.0, *p* < 0.05) (Figure 3D).

### SWE affects motility of *S*. Enteritidis

**Figure 4:**
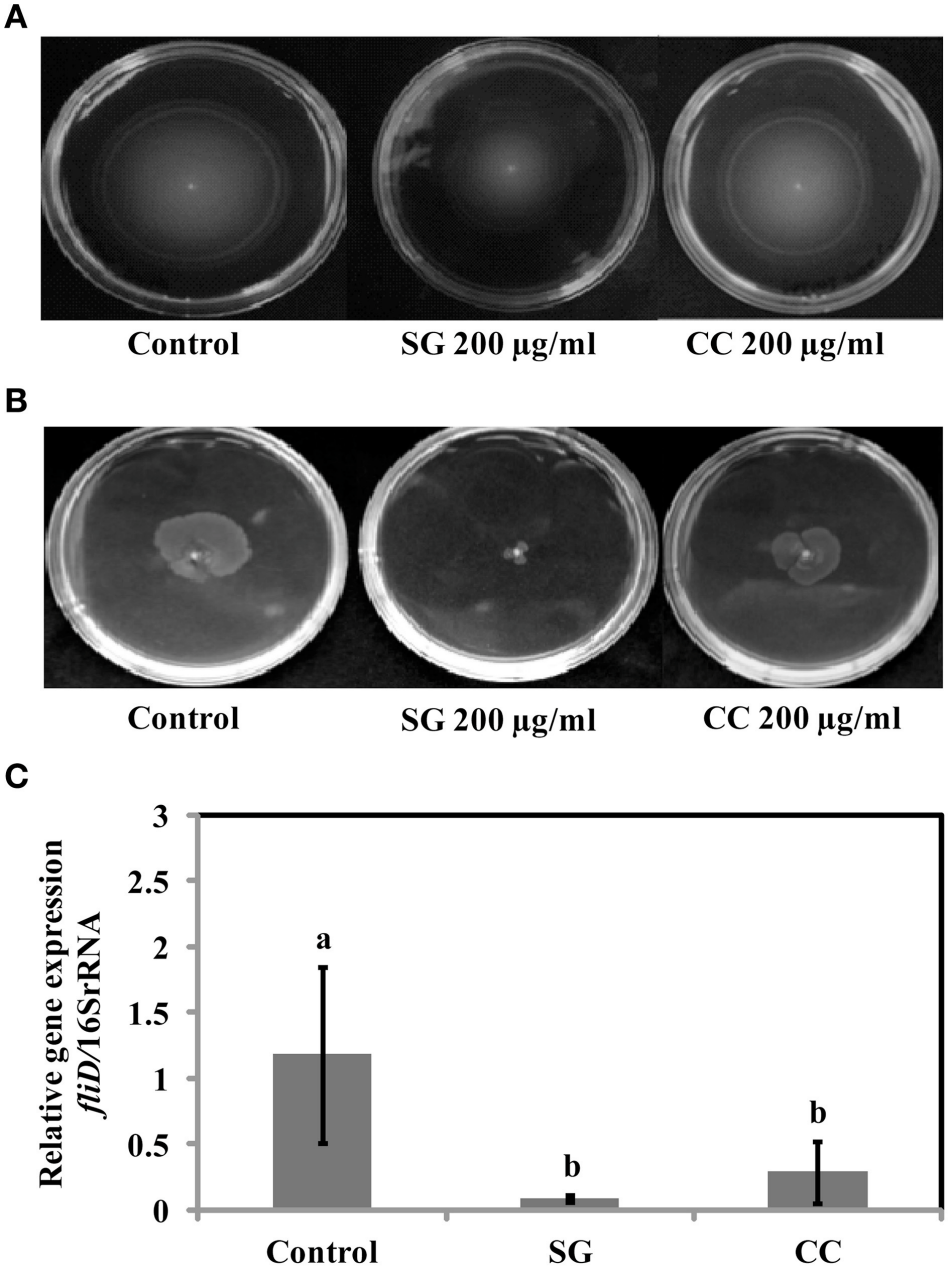
**Effect of SWE on the motility of bacteria. (A)** SWE effect on *S*. Enteritidis swimming motility. Swimming motility of *S*. Enteritidis was determined by adding 200 μl/ml SWE into the agar plates. Single purified colony was inoculated with a toothpick from an overnight TSA plate onto a swim plate (tryptone broth plus 0.3% agar) to observe for effect on motility after overnight incubation at 30°C. **(B)** SWE effect on *S*. Enteritidis swarming motility. *S*. Enteritidis showed deficient movement when inoculated onto swarm plates (Difco bacto-agar, 0.5% glucose) after 24 h incubation at 30°C. **(C)** Effect of SWE on the relative gene expression of fliD gene required for polymerization of flagellin on the tip of growing flagella. Values represent Mean ± Standard deviation from three independent experiments; each experiment had three biological replicates.

*Salmonella* motility plays a key role in the initial establishment and colonization of host tissues. Therefore, we tested if SWE affect S. Enteritidis motility. Motility tests were performed as described by Rashid and Kornberg (2000) with some modifications. Compared to control, amending SG (200 μg/ml) water extract showed a significantly reduction (70–90%) in swimming (helical rotation of flagella in liquid agar plates) and swarming (multicellular translocation on semisolid agar plates) motility (Figures 4A,B). However, CC (200 μg/ml) water extract did not significantly affect the motility of S. Enteritidis compared to SG or control (Figures 4A,B). CC water extract reduced the swimming motility by 5–10% (Figure 4A) and swarming motility by 20–25% (Figure 4B) compared to control plates.

### SWE inhibits biofilm formation of SE

**Figure 5:**
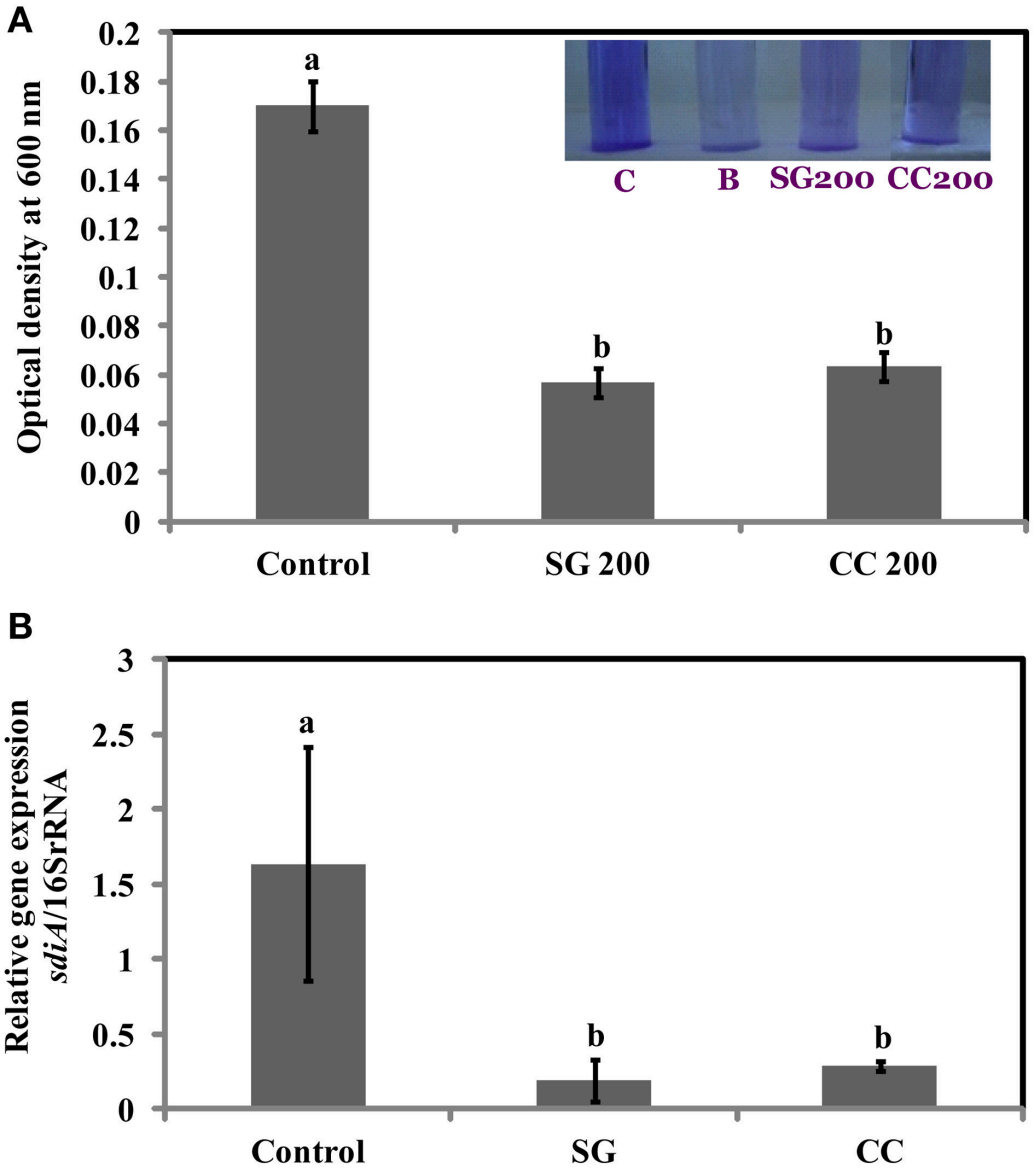
**Effect of SWE treatment on biofilm formation of *S*. Enteritidis**. *S*. Enteritidis culture was statically grown for 24 h at 37°C in polyvinyl chloride microtitre plates in the presence of 200 μl/ml SWE (SG or CC). **(A)** Biofilm formation was quantified by staining with crystal violet and determining the optical density at 600 nm. **(B)** Effect of SWE on the relative gene expression of *sdiA* gene (homolog of quorum-sensing regulators LuxR). Values with different superscript letters (Tukey multiple mean comparison) are significantly different (one-way Anova; *p* < 0.05). Values represent Mean ± Standard deviation from three independent experiments; each experiment had three biological replicates. Picture insert: C, positive control; B, negative control; SG200, 200 μl/ml SG extract; CC200, 200 μl/ml CC extract.

Biofilms are increasing the survival of bacteria in adverse environmental conditions and contributes to the virulence. As motility regulates biofilm formation, we tested if SWE reduces biofilm formation by *S*. Enteritidis. Addition of SWE (200 μg/ml, CC and SG) significantly decreased (*p* < 0.05*)* biofilm formed by *S*. Enteritidis (Figures 5A,B). Presence of CC and SG in the culture medium resulted in biofilm formation equivalent to an optical density of 0.06 ± 0.004 and 0.05 ± 0.005 respectively, which is 3–4-fold lower when compared to control (OD_600_ = 0.17 ± 0.01) (Figures 5A,B).

### SWE suppress the expression of virulence and quorum sensing related genes in *s*. Enteritidis

**Figure 6:**
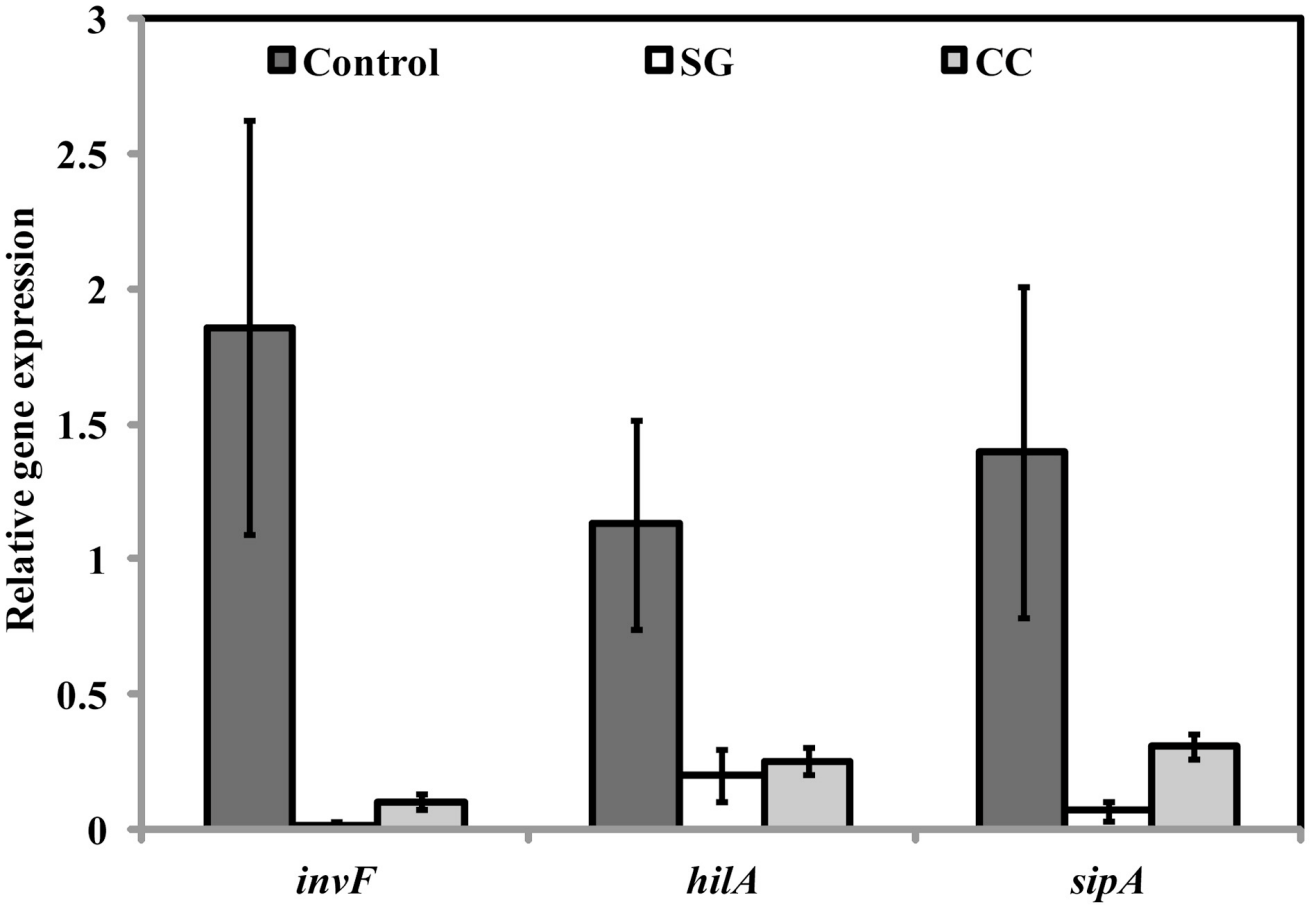
**Effect of SWE on the relative expression of virulent genes**. SPI-1 encodes genes (*sdiA, hilA, fliD, sipA*, and *invF*) required for the invasion of *S*. Enteritidis into the host epithelium (Type 3 secretion system). Values with different superscript letters are significantly different (Tukey multiple mean comparison, *p* < 0.05). Values represent Mean ± Standard deviation from three independent experiments; each experiment had three biological replicates.

Virulence factors including type 3 secretion system, filaments, and flagella are required for the initial attachment and subsequent internalization of *S*. Enteritidis in the intestinal epithelium. *S*. Enteritidis regulates the gene expression patterns in response to changes in population density by quorum sensing. Therefore, the effect of SWE (CC and SG) on the expression of virulence and quorum sensing genes was determined. SWE (CC and SG) suppressed the expression of genes without affecting the housekeeping genes, *tufA* and *16S rRNA* (Figures 4C, 5B, 6). The relative expression of quorum sensing transcriptional activator *sdiA* which encodes for SdiA (LuxR homolog), *Salmonella* pathogenicity island-1 (SPI-1) regulator *hilA* and flagellar hook associated *fliD* genes were repressed 4–13 times (*p* < 0.001). Similarly, SPI-1 associated genes (*sipA* and *invF*) were down regulated 16–20 times by SG water extract, and 4–8 times by CC water extract (*p* < 0.001) (Figure 6), respectively. This suggests that SWE might reduce the invasion of *S*. Enteritidis in the host by attenuating virulence factors and quorum sensing.

### SWE protects *C. elegans* from infection by *S*. Enteritidis

**Figure 7:**
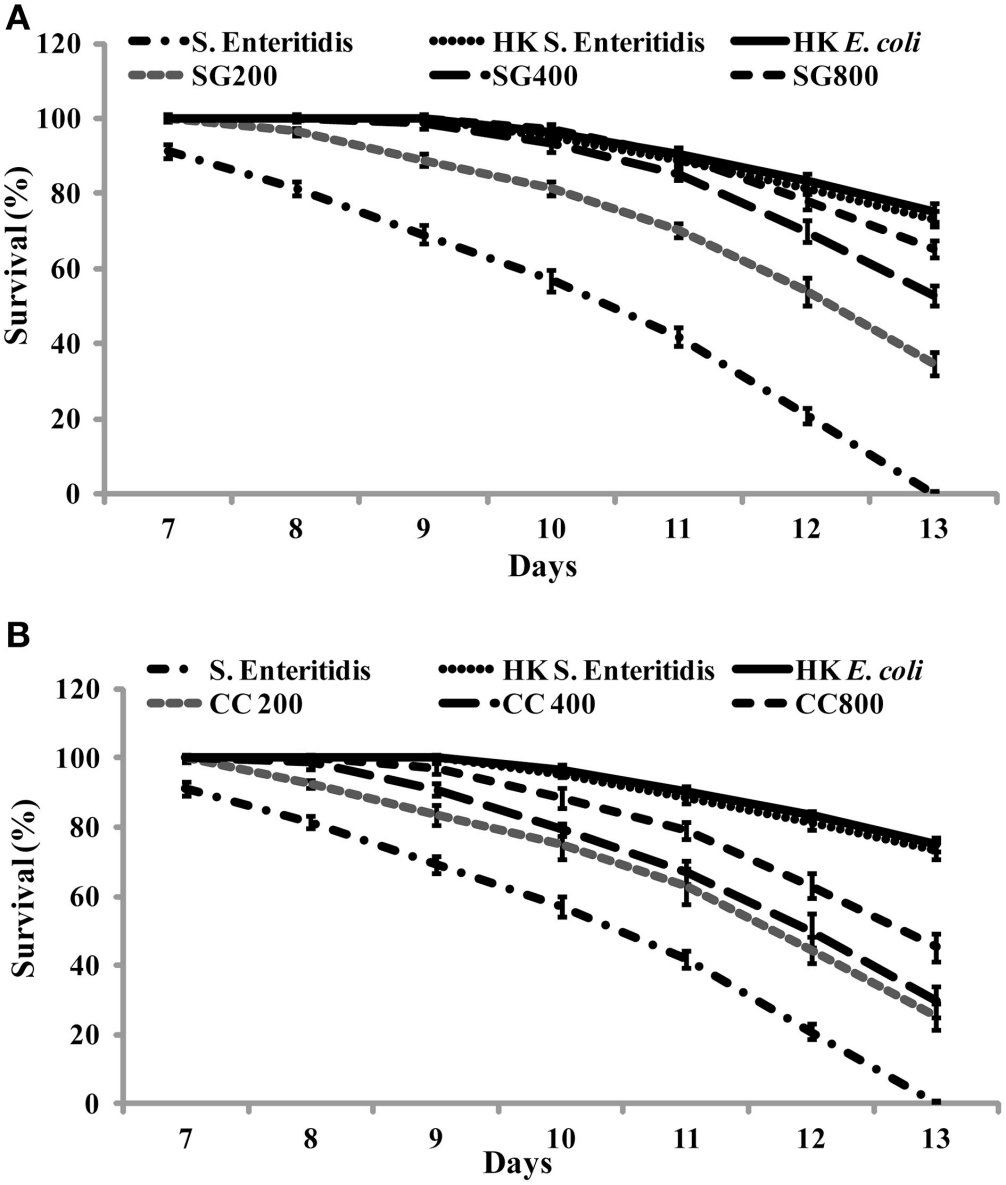
**The effect of SWE on the survival of nematodes infected with *S*. Enteritidis**. Three concentrations of SWE (200, 400, and 800 μg/ml) were used with. Worms were grown with seaweed water extract as food supplements and were exposed to *S*. Enteritidis. **(A)** SG treatment and **(B)** CC treatment. Values represent Mean ± Standard deviation from two experiments with six biological replicates.

As CC and SG seaweed extracts exhibited antimicrobial activity *in vitro*, we tested their effect on *C. elegans*. Under experimental conditions, 13 ± 1 days were required for L4 stage nematodes to be killed by *S*. Enteritidis colonization. *C. elegans* were cultured on *E. coli* OP50 as food source and SWE (CC and SG) were added to the food source from early L1 stage. On day 1 of L4 stage, the worms were exposed to *S*. Enteritidis in the presence and absence of SWE (CC and SG; 200, 400, 800 μg/ml). The SWE were added to the culture medium and the food source on NGM plates. The survival percentages of the worms were recorded each day and infected worms without seaweed supplement served as control. SWE (CC and SG) increased the survival percentage of the worms, when used either as food source for worms (pre-treatment of worms with SWE) or as an inhibitor of *S*. Enteritidis in the culture medium (pre-treatment of bacteria with SWE) (data not shown). However, the combination treatment (pre-treatment of bacteria and worms with SWE) showed the highest rates of protection of the worms from *S*. Enteritidis infection (Figure 7). For the SG water extract, the percentage survival on day 13 was increased (*p* < 0.0001) by 35% at 200 μg/ml, 53% at 400 μg/ml and 65% at 800 μg/ml (Figure 7A). Likewise, CC water extract also resulted in survival rates higher than control. On day 13 the CC water extract increased the survival of *S*. Enteritidis infected worms by 25, 29, and 45% with treatments of 200, 400, and 800 μg/ml, respectively (Figure 7B), respectively. Compared to control, both CC and SG water extract significantly increased the survival of the infected worms, however treatment in pairwise comparisons for all concentrations SG showed higher survival rates (*p* < 0.0001). Moreover, no significant differences were observed in the application of SWE incorporated either into the medium (data not shown) or over the NGM growth medium (data not shown). *C. elegans* did not show any developmental abnormalities (such as cell death abnormality, egg hatching defects, abnormal body size and small, thin and slow growth phenotype) when fed with SWE along with food source *E coli* OP50 and the rate of feeding was uniform in all treatments.

### SWE reduces accumulation of *S*. Enteritidis in *C. elegans*

**Figure 8:**
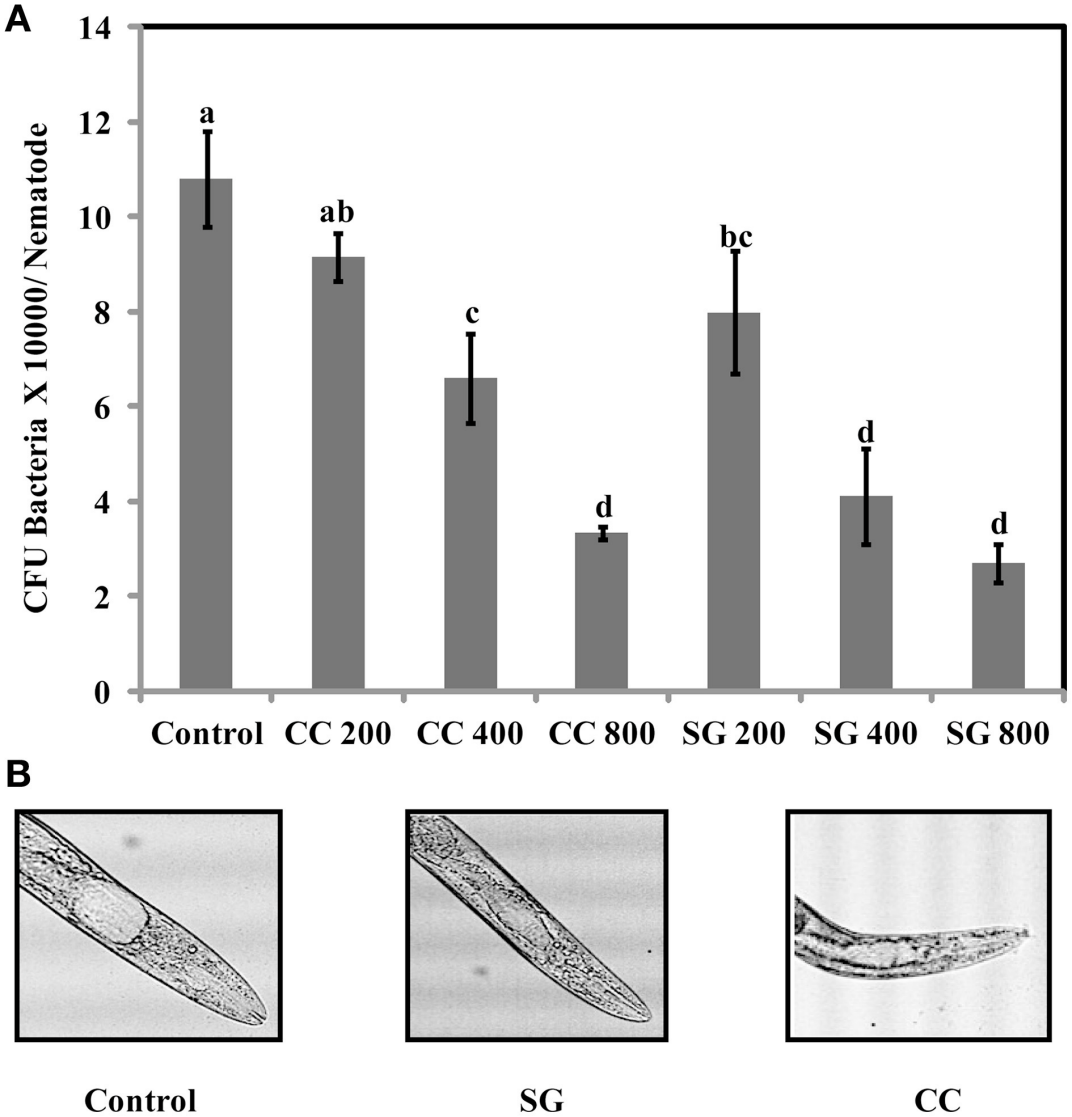
***S*. Enteritidis colony counts in the gut of *C. elegans***. **(A)** Effect of SWE on bacterial CFU count in the *C. elegans* gut. **(B)** Microscopic images showing the gut of *C. elegans* infected with *S*. enteritidis. In control the gut is swelled while much less swelling was observed when infected nematodes were treated with SWE. Values with different superscript letters are significantly different (Tukey multiple mean comparison, *p* < 0.05). Values represent Mean ± Standard deviation from two experiments with six biological replicates.

The increase in survival of *S*. Enteritidis infected *C. elegans* by SWE supplementation could be due to an impaired ability of *S*. Enteritidis to colonize the digestive tract of nematode. Hence, the number of viable bacteria in *C. elegans* was enumerated by standard plate count method. SG was found to be slighter more effective than CC. All concentrations of SG, that is 200, 400, and 800 μg/ml of SG water extract were effective in reducing the colony count (CFU ^*^10,000 = 7.9, 4.1, and 2.7) of *S*. Enteritidis in the worms. For CC water extract, only 400 and 800 μg/ml were significantly effective (CFU ^*^10,000 = 6.5 and 3.3) in reducing the bacterial CFU in the gut of *C. elegans* when compared to control (Figure 8).

### SWE induces immune response genes in *S*. Enteritidis infected and non-infected *C. elegans*

**Figure 9:**
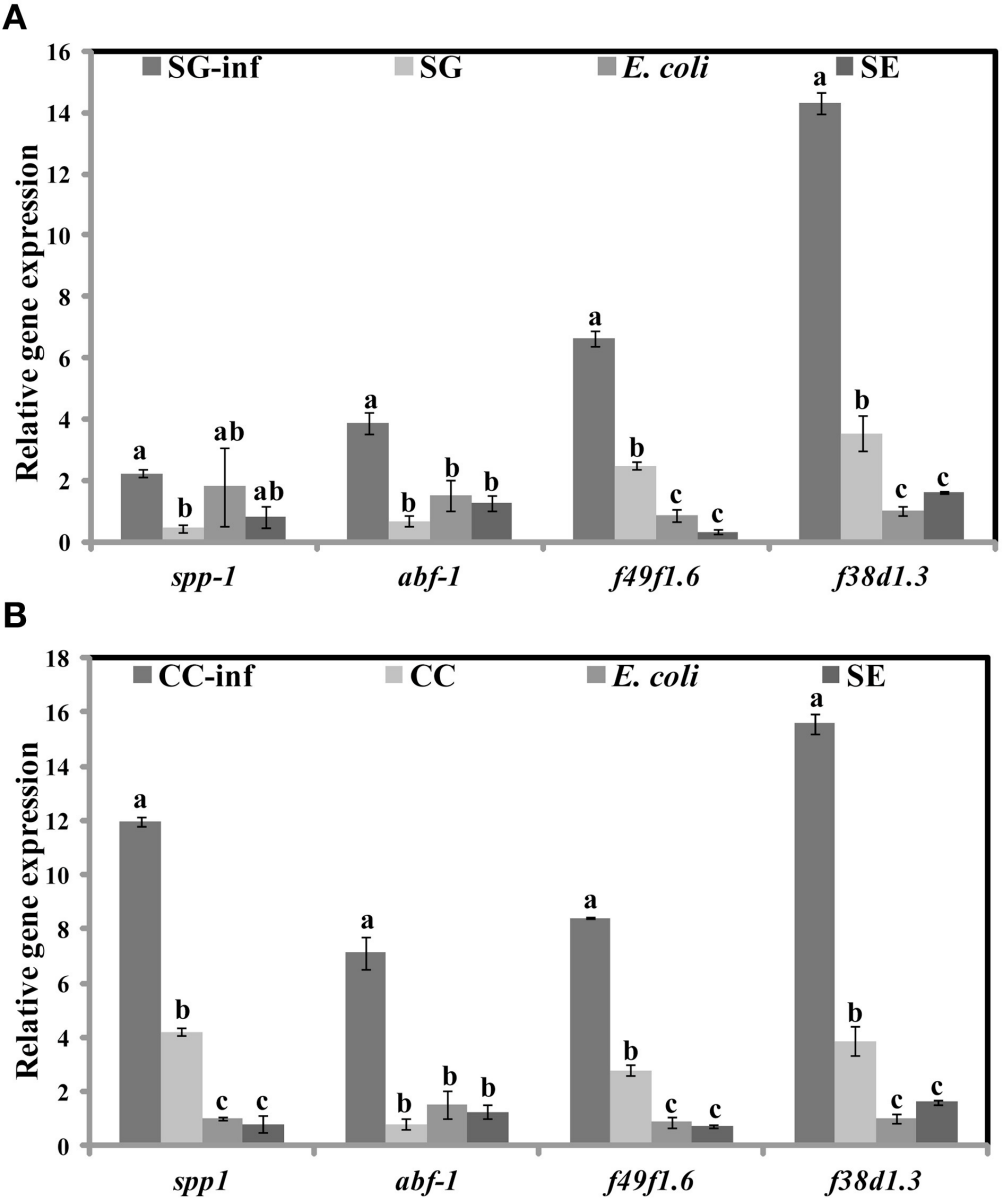
**Effect of SWE on the relative expression of immune responsive genes**. Expression of the immune response genes *spp-1* (saponin like protein), *abf-1* (antibacterial protein), *f49f1.6* (ShK domain-like, PMK-1) and *f38a1.5* (lectin family protein) after 5 days of exposure to **(A)** SG or **(B)** CC was analyzed by quantitative reverse transcription PCR. SG**-**inf, *C. elegans* fed on SG and infected with *S*. Enteritidis; SG, *C. elegans* fed on SG; *E. coli, C. elegans* fed on heat killed *E. coli*; SE, *C. elegans* fed on *S*. Enteritidis. Values with different superscript letters are significantly different (Tukey multiple mean comparison, *p* < 0.05). Values represent Mean ± Standard deviation from three independent experiments; each experiment had three biological replicates.

Apart from the inhibition of virulence factors, another mechanism by which SWE can increase the survival of *C. elegans*, following *S*. Enteritidis exposure, is by enhancing the immune system of the nematode. Therefore, we tested the effect of SWE (SG and CC) on the expression of *C. elegans* immune response genes by quantitative reverse transcription PCR. Both SG and CC extracts up-regulated *f49f1.6* (ShK domain-like, PMK-1) and *f38a1.5* (lectin family protein) while CC also increased the expression of *spp-1* (saponin like protein) gene. Five days after the infection with *S*. Enteritidis most of the tested immune genes were up-regulated in the SWE (SG and CC)-treated *S*. Enteritidis-infected *C. elegans* (i.e., nematodes fed on SWE and exposed to *S*. Enteritidis) but not in *C. elegans* fed on *S*. Enteritidis alone. In the presence of the SG extract the immune response genes *spp-1, abf-1* (antibacterial protein), *f49f1.6* and *f38a1.5* were 1.5–15 times fold up-regulated vs control (nematodes fed on heat killed *E. coli*). Similarly, all immune response genes analyzed in CC-treated *S*. Enteritidis-infected *C. elegans* were up-regulated compared to control: *spp-1* was induced 12 times, *abf-1* 6 times, *f49f1.6* 7 times while *f38a1.5* 17 times; all these differences were found to be statistically significant (Figure 9B). Overall, the level of activation of expression of the four immune genes was higher in the presence of CC compared to that observed in the presence of SG (Figures 9A,B).

## Discussion

In this study, we report the antimicrobial activity of the cultivated red seaweeds, *Chondrus crispus* and *Sarcodiotheca gaudichaudii*, against the enteric pathogen *Salmonella* Enteritidis. Water extract of CC and SG reduced biofilm formation, motility and down regulated the expression of genes encoding the virulence factors of *S*. Enteritidis. Moreover, the extracts also protected the soil nematode *C. elegans* from killing by *S*. Enteritidis. In growth inhibition assay, the higher concentrations of CC extract (1 and 2 mg/ml) were the most effective in reducing the growth of *S*. Enteritidis, likely showing a dose dependent response of CC water extract on the reduction of the colony count of *S*. Enteritidis. However, for SG, although higher concentrations (1 and 2 mg/ml) significantly reduced bacterial titers, an increased in colony count was observed with 2 mg/ml compared to 1 mg/ml. This indicates that the threshold concentration of antimicrobial compounds in the crude extract could be less than 2 mg/ml. Red seaweeds are rich in sugars such as D-galactose and D-glucose (Kim, 2011). Thus, these sugars present in the extracts can serve as a carbon source at higher concentrations (<2 mg/ml) that could enhance the growth of *S*. Enteritidis.

After entering the host by oral route, *S*. Enteritidis uses several strategies to colonize and persist in the cell. *S*. Enteritidis utilizes flagellar motility including swimming and swarming to move toward the favorable environment for colonization and produce biofilm and virulence factors to spread pathogenicity (Bogomolnaya et al., 2014). Previous studies have shown a strong correlation between biofilm formation, antimicrobial resistance and persistent infection (Wang et al., 2013). *Salmonella* adhere and survive on surfaces for a prolonged period by forming biofilms. Biofilms protect *Salmonella* from several stresses including antimicrobials, temperature and the host immune system. Several chemical agents such as sodium hypochlorite, alkaline peroxide and benzalkonium chloride are commonly used to eliminate *Salmonella* biofilms. However, limitations such as corrosion, toxicity and development of microbial resistance, restrict the use of such compounds. Hence, alternative strategies are required to interfere with the *Salmonella* biofilm formation. Our results showed that SWE (CC and SG) effectively reduced the buildup of biofilm by *S*. Enteritidis on polystyrene at a concentration (200 μg/ml) that did not affect the growth of planktonic cell. This could be due to the presence of quorum sensing inhibitors in the SWE. Quorum sensing (QS), cell-to-cell bacterial communication system involves signaling molecules such as autoinducers (acylated homoserine lactones, AHL). Quorum sensing mediates virulence, motility and biofilm formation in human pathogens including *Salmonella* (Jesudhasan et al., 2010; Choi et al., 2012). Previously, halogenated furanones of red algae *Delisea pulchra* were shown to be an antagonist of AHL mediated gene expression. The furanones inhibited quorum sensing in gram negative bacteria by interfering with AHL mediated gene expression of LuxR protein (Manefield et al., 1999). Similarly, synthetic furanones improved *P. aeruginosa* clearance from lungs in mice by inhibiting bacterial quorum sensing (Wu et al., 2004). Janssens et al. (2008) identified brominated furanones of *Delisea pulchra*, capable of inhibiting *S. enterica* serovar Typhimurium biofilm formation. They concluded that the expression of target gene of quorum sensing system such as AHL receptor SdiA was not altered on treatment with brominated furanones. Biofilm formation was reduced by repressing the expression of the global flagellar regulator *flhD* (Janssens et al., 2008). In the present study, expression of *sdiA* (activator of SdiA) was repressed by both CC and SG. The algal extracts likely impaired quorum sensing and thus contributed to reduced biofilm formation. However, the specific seaweed compounds responsible for repressing quorum-sensing regulators remain to be identified.

Presence of functional flagella is required for both biofilm formations and motility in gram-negative bacteria. Depending on environmental conditions, flagella are involved in initial reversible attachment as well as release of motile cells from mature biofilm (Chelvam et al., 2014). In present study, SWE (200 μg/ml, CC and SG) reduced the swimming and swarming motility of *S*. Enteritidis. Additionally, SWE also down-regulated the expression of *fliD* gene that is required for polymerization of flagellin. This indicates that restricted *S*. Enteritidis motility in the presence of SWE could be due the inability of flagellin molecules to assemble onto the hook (Yokoseki et al., 1995). Although both SWE (CC and SG) reduced *S*. Enteritidis motility, cells treated with SG extracts formed smaller swarms on motility agar plates. This could be due to the combined effects of SG on both, flagellar biosynthesis and quorum sensing (Manefield et al., 1999; Janssens et al., 2008). Flagellar based motility contributes to the virulence of pathogen through adhesion, biofilm formation and translocation of virulent protein via Type 3 secretion system. Type 3 secretion system enables *S*. Enteritidis to invade and survive within the cell (Galan, 2001). SWE (CC and SG) reduced the expression of SP-1 encoded virulent factors *hilA, invF*, and *sipA*. A previous study showed that obacunone, a triterpenoid from citrus, repressed SP-1 of *Salmonella enterica serovar* Typhimurium mediated through *hilA* (Vikram et al., 2012). Another study revealed that *Chondrus crispus* water extract reduced the virulence factors and QS genes in *Pseudomonas aeruginosa* (PA-14) (Liu et al., 2013). Interestingly, in both the studies, the extracts did not show a direct effect on the growth of bacteria. However, in the present study, higher concentrations (800 μg/ml, 1 and 2 mg/ml) of SG and CC extract showed direct antimicrobial effect. The growth inhibition activity might be due to an alteration of the cell wall integrity of *S*. Enteritidis. Higher concentrations of the extracts likely changed the permeability of the cell, resulting in cell lysis and leakage of intracellular content (Hierholtzer et al., 2013).

In the present study, we used *C. elegans* infection model to investigate the effects of SWE on *S*. Enteritidis pathogenicity and worm innate immunity. SWE (CC and SG) significantly increased the survival of *C. elegans* infected with *S*. Enteritidis. Additionally, highest concentration of SWE (800 μg/ml CC and SG), significantly reduced the accumulation of *S*. Enteritidis in *C. elegans* gut. The reduced *S*. Enteritidis colonization could be partially due to the decrease in the ability of bacterial to attach to the surface of the intestinal epithelium of *C. elegans* (Aballay et al., 2000). The reduction in bacterial attachment might be due to the affect of SWE on biosynthesis of flagellar components. Compared to control, low concentration of SWE (200 μg/ml CC and SG), significantly improved the survival of the worms, however, it did not affect the population of bacteria in the gut. This data suggest that low concentrations of CC and SG SWE increased the survival of nematodes by repressing the expression of SP-1 genes *hilA* and *infF* that are essential for virulence and killing in *C. elegans* (Tenor et al., 2004). *C. elegans* immune responses up-regulate the expression of defense related genes required to combat the infection caused by invading pathogens, including *Salmonella* (Alegado and Tan, 2008). In the present study, in *C. elegans* infected with *S*. Enteritidis, both CC and SG water extracts were found to induce the expression of immune related genes *f49f1.6* (regulated by the PMK-1), *spp-1, abf-1*, and lectin family protein *f38a1.5*. The level of expression of these genes was higher in the presence of CC compared to SG water extract indicating a stronger effect of CC extract on *C. elegans* immune system. Notably, the expression of immune related genes was also induced without infection, indicating that SWE can augment immune responses in *C. elegans* (Liu et al., 2013).

In conclusion, SWE (CC and SG) inhibited the growth, motility and biofilm formation of *S*. Enteritidis. Furthermore, gene expression analysis showed that SWE inhibited the quorum sensing, virulence and motility related genes. This indicates that a possible mechanism of *S*. Enteritidis inhibition by SWE could be by interfering with flagellar biosynthesis. Another possibility is that SWE inhibit quorum sensing through compounds that are structurally similar to auto-inducers. Additionally, SWE reduced *S*. Enteritidis colonization in *C. elegans* and increased the survival of infected worms. Both CC and SG increased the survival of *C. elegans*; however, the mode of action and the level of activity appear to be different. SG was more effective as an antimicrobial, reducing *S*. Enteritidis invasion, whereas CC stimulated more the immune responsive genes, enhancing *C. elegans* immunity and, thereby, increasing their survival. Further studies are required to provide more support to these findings and to contribute to the understanding of the inhibitory mode of action of SWE on *S*. Enteritidis. Moreover, additional investigations are needed to identify and isolate the compound(s) responsible for the antibacterial activity. Taken together, our data indicate that CC and SG water extracts have significant antimicrobial effects on *S*. Enteritidis, improving the survival of *S*. Enteritidis-infected *C. elegans*.

## Author contributions

Conceived and designed the experiments: GK and BP. Performed the experiments, contributed with reagents, biological materials or assisted with data acquisition, analysis and interpretation: GK, TB, BR, GS, NT, AC, JH, and BP. Drafted the manuscript: GK, TB, BR, GS, NT, AC, JH, and BP. All authors critically revised and approved the final version.

### Conflict of interest statement

The authors declare that the research was conducted in the absence of any commercial or financial relationships that could be construed as a potential conflict of interest.
