# Supplementary material for: Red Seaweeds Sarcodiotheca gaudichaudii and Chondrus crispus down Regulate Virulence Factors of Salmonella Enteritidis and Induce Immune Responses in Caenorhabditis elegans
Source: Front Microbiol. 2016 Mar 31;7:421. doi: 10.3389/fmicb.2016.00421 (PMC4814495; doi:10.3389/fmicb.2016.00421)
Supplement: Supplementary file 1 [file Table1.DOCX]

Supplementary Material

**Red seaweeds *Sarcodiotheca gaudichaudii* and *Chondrus crispus* down regulate virulence factors of *Salmonella* Enteritidis and induce immune responses in *Caenorhabditis elegans***

**Garima Kulshreshtha^1,4^, Tudor Borza^1^, Bruce Rathgeber^2^, Glenn Stratton^1^, Nikhil Thomas^3^, Alan Critchley^4^, Jeff Hafting^4^ and Balakrishnan Prithiviraj^1#*^**

^1^Department of Environmental Sciences, Faculty of Agriculture, Dalhousie University, PO Box 550, Truro, NS, Canada, B2N 5E3

^2^Department of Plant and Animal Sciences, Faculty of Agriculture, Dalhousie University, PO Box 550, Truro, NS, Canada, B2N 5E3

^3^Department of Microbiology and Immunology, Faculty of Medicine, Dalhousie University, Halifax, NS, Canada B3H 4J1

^4^Acadian Seaplants Limited, 30 Brown Avenue, Dartmouth, NS, Canada. B3B 1X8

**Correspondence:** Balakrishnan Prithiviraj, Department of Environmental Sciences, Faculty of Agriculture, Dalhousie University, PO Box 550, Truro, NS, Canada, B2N 5E3, bprithiviraj@dal.ca; Tel: +1 902 893 6643; Fax: +1 902 895 6734

# Supplementary Table 1. *S.* Enteritidis genes and primer sequences used for amplification by RT-qPCR

| **Gene** |  | **Primer Sequence (5′ → 3′)** |
| --- | --- | --- |
| *16S rRNA* | Fw  Rev | GCGGCAGGCCTAACACAT  GCAAGAGGCCCGAACGTC |
| *tufA* | Fw  Rev | TGTTCCGCAAACTGCTGGACG  ATGGTGCCCGGCTTAGCCAGTA |
| *sipA* | Fw  Rev | CCAACGCAATGGCGAGTCAC  GCCGTCTCCGTTTGATGCGT |
| *fliD* | Fw  Rev | TCACCACCAAAATTGCCACC  CCTTGTAACGGGCAACGGT |
| *invF* | Fw  Rev | TTTGCGAGCAGGCCGTTGTC  GCGCCATCGATAAATGCCAGT |
| *hilA* | Fw  Rev | GGTTTAATCGTCCGGTCGTAGTG  CCTGATCCTGCATCTGAAAAGG |
| *sdiA* | Fw  Rev | GCCGCCCAGCGTTTCGGATT  AAAAGCCCAGCGCCCGGTTC |
