# Supplementary material for: Red Seaweeds Sarcodiotheca gaudichaudii and Chondrus crispus down Regulate Virulence Factors of Salmonella Enteritidis and Induce Immune Responses in Caenorhabditis elegans
Source: Front Microbiol. 2016 Mar 31;7:421. doi: 10.3389/fmicb.2016.00421 (PMC4814495; doi:10.3389/fmicb.2016.00421)
Supplement: Supplementary file 2 [file Table2.DOCX]

Supplementary Material

**Red seaweeds *Sarcodiotheca gaudichaudii* and *Chondrus crispus* down regulate virulence factors of *Salmonella* Enteritidis and induce immune responses in *Caenorhabditis elegans***

**Garima Kulshreshtha^1,4^, Tudor Borza^1^, Bruce Rathgeber^2^, Glenn Stratton^1^, Nikhil Thomas^3^, Alan Critchley^4^, Jeff Hafting^4^ and Balakrishnan Prithiviraj^1#*^**

^1^Department of Environmental Sciences, Faculty of Agriculture, Dalhousie University, PO Box 550, Truro, NS, Canada, B2N 5E3

^2^Department of Plant and Animal Sciences, Faculty of Agriculture, Dalhousie University, PO Box 550, Truro, NS, Canada, B2N 5E3

^3^Department of Microbiology and Immunology, Faculty of Medicine, Dalhousie University, Halifax, NS, Canada B3H 4J1

^4^Acadian Seaplants Limited, 30 Brown Avenue, Dartmouth, NS, Canada. B3B 1X8

**Correspondence:** Balakrishnan Prithiviraj, Department of Environmental Sciences, Faculty of Agriculture, Dalhousie University, PO Box 550, Truro, NS, Canada, B2N 5E3, bprithiviraj@dal.ca; Tel: +1 902 893 6643; Fax: +1 902 895 6734

Supplementary Table 2. The *C. elegans* immune responsive genes name and primer sequences used in RT-qPCR

| **Gene** | |  | **Primer Sequence (5′ → 3′)** |
| --- | --- | --- | --- |
| *f49f1.6* | Fw  Rev | | TGCACTACTACATCCTGCCTATTC  CCGGACATGTGATCATTGAG |
| *spp-1* | Fw  Rev | | TGAACATCGGAACTCTTTGC  TCAGCTCTTCCTCACACTCG |
| *f38d1.3* | Fw  Rev | | CTGGGCCGGTATTAATTTGT  GTCTTCTTCGTCACGCACAT |
| *abf-1* | Fw  Rev | | TGCCTTCTCCTTGTTCTCCT  ATCCTCTGCATTACCGGAAC |
| *Ama-1* | Fw  Rev | | CTGACCCAAAGAACACGGTGA  TCCAATTCGATCCGAAGAAGC |
