# Supplementary material for: Red Seaweeds Sarcodiotheca gaudichaudii and Chondrus crispus down Regulate Virulence Factors of Salmonella Enteritidis and Induce Immune Responses in Caenorhabditis elegans
Source: Front Microbiol. 2016 Mar 31;7:421. doi: 10.3389/fmicb.2016.00421 (PMC4814495; doi:10.3389/fmicb.2016.00421)
Supplement: Supplementary file 3 [file Table3.DOCX]

Supplementary Material

**Red seaweeds *Sarcodiotheca gaudichaudii* and *Chondrus crispus* down regulate virulence factors of *Salmonella* Enteritidis and induce immune responses in *Caenorhabditis elegans***

**Garima Kulshreshtha^1,4^, Tudor Borza^1^, Bruce Rathgeber^2^, Glenn Stratton^1^, Nikhil Thomas^3^, Alan Critchley^4^, Jeff Hafting^4^ and Balakrishnan Prithiviraj^1#*^**

^1^Department of Environmental Sciences, Faculty of Agriculture, Dalhousie University, PO Box 550, Truro, NS, Canada, B2N 5E3

^2^Department of Plant and Animal Sciences, Faculty of Agriculture, Dalhousie University, PO Box 550, Truro, NS, Canada, B2N 5E3

^3^Department of Microbiology and Immunology, Faculty of Medicine, Dalhousie University, Halifax, NS, Canada B3H 4J1

^4^Acadian Seaplants Limited, 30 Brown Avenue, Dartmouth, NS, Canada. B3B 1X8

**Correspondence:** Balakrishnan Prithiviraj, Department of Environmental Sciences, Faculty of Agriculture, Dalhousie University, PO Box 550, Truro, NS, Canada, B2N 5E3, bprithiviraj@dal.ca; Tel: +1 902 893 6643; Fax: +1 902 895 6734

Supplementary Table 3. Antibacterial activity of crude seaweed extracts on *S.* Enteritidis

| Concentration  (mg/well) | **Seaweeds (Growth inhibition) ^1^** | |
| --- | --- | --- |
|  | *Chondrus crispus* | *Sarcodiotheca gaudichaudii* |
| 0.2 | 0 | 0 |
| 0.4 | 0 | 3.67±0.33 |
| 0.8 | 0 | 4.83±0.21 |
| 1.0 | 4.08±0.16 | 8.42±0.30 |
| 1.6 | 7.85±0.90 | 10.49±0.19 |
| 2.0 | 9.40±0.26 | 13.22±0.52 |

^1^ Diameter of zone of growth inhibition in mm

Values represent mean ± standard deviation from three independent experiments; each experiment had three biological replicates
